# Testing reward‐cue attentional salience: Attainment and dynamic changes

**DOI:** 10.1111/bjop.12537

**Published:** 2021-10-28

**Authors:** Matteo De Tommaso, Massimo Turatto

**Affiliations:** ^1^ Center for Mind/Brain Sciences University of Trento Trent Italy

**Keywords:** attention, devaluation, reward, salience, selection history

## Abstract

A great wealth of studies has investigated the capacity of motivationally relevant stimuli to bias attention, suggesting that reward predicting cues are prioritized even when reward is no longer delivered and when attending to such stimuli is detrimental to reward achievement. Despite multiple procedures have been adopted to unveil the mechanisms whereby reward cues gain attentional salience, some open questions remain. Indeed, mechanisms different from motivation can be responsible for the capture of attention triggered by the reward cue. In addition, we note that at present only a few studies have sought to address whether the cue attractiveness dynamically follows changes in the associated reward value. Investigating how and to what extent the salience of the reward cue is updated when motivation changes, could help shedding light on how reward‐cues attain and maintain their capacity to attract attention, and therefore on apparent irrational attentive behaviors.

## INTRODUCTION

Motivationally relevant objects attract attention (e.g., Engelmann & Pessoa, 2007). However, in the last two decades a great bulk of evidence has accumulated showing that through different learning mechanisms also reward predicting cues become capable of summoning visual attention (Anderson, 2016a; Chelazzi et al., 2013; Failing & Theeuwes, 2017b; Le Pelley et al., 2016). The reward‐cue attentional salience has been shown to be determined by the associated reward value, and importantly to persist for some time during extinction, when reward is no longer delivered (Anderson et al., 2011; Anderson & Yantis, 2013). This scenario is in general agreement both with the classic conditioning models assuming that reward‐predicting stimuli attract attention because of their information content (Mackintosh, 1975; Pearce & Hall, 1980), and with more motivation‐based models which postulate that during Pavlovian conditioning the motivational and attentional salience of the unconditioned stimulus (US) transfer to the conditioned stimulus (CS), which then becomes an attentional magnet (Berridge, 2018; Bindra, 1978; Toates, 1986).

Although there is a general consensus on the idea that the reward‐cue attentional salience is modulated by the associated reward value, less obvious is whether once acquired the cue salience varies when reward value changes over time. For example, since the motivational value of palatable rewards fluctuates as a function of the physiological level of the corresponding drive (e.g., hunger or thirst), the question is whether the reward‐cue attentional salience does also change accordingly. Intuitively this should be the case, but the study of De Tommaso et al. (2017) has shown that the attentional salience of a reward cue can outlast reward devaluation, a result that indicates that the cue salience is not automatically updated when reward value changes. Such lack of flexibility may have important implications for our understanding of the learning mechanisms governing the interplay between attention and motivation, and could also partially explain the occurrence of an apparently irrational phenomenon that is evident in addiction. Indeed, addicts seem to be constantly attracted by drug cues far beyond the symptoms of abstinence have ceased, and despite the drug is neither explicitly wanted nor would be pleasantly consumed (Berridge & Robinson, 2016), a circumstance resembling a dissociation between the cue incentive salience and its associated reward value.

However, concerns have been raised that some of the reported evidence of the reward‐cue attentional bias may in fact result from factors other than motivation, like for instance strategic attentional allocation or selection history (Anderson & Halpern, 2017; Awh et al., 2012; Failing & Theeuwes, 2017b; Marchner & Preuschhof, 2018; Mine & Saiki, 2015; Sha & Jiang, 2016). For example, certain perceptual characteristics of reward associated cues can be explained by top‐down attentional control when reward gaining is the goal of the task (Maunsell, 2004; Sawaki et al., 2015). In this case, attention to reward‐cues would be strategically enhanced because of reward prospect. However, an attentional bias driven by strategic mechanisms is not informative about the dynamic changes in reward value because such strategic behaviour will subside when reward is not the task goal. Additionally, an alternative explanation to motivation is that the reward‐cue salience can be determined by past episodes of attentional selection. When the attentional processing of the cues correlates with reward delivery, the effects of selection history and the effects due to the motivational component of reward are indistinguishable. Hence, an ideal paradigm to detect reward‐driven modulation of the cue attentional salience, including its dynamic changes, if any, must be capable of ruling out alternative explanations. It is therefore worth analysing the paradigm used in studies showing the persistence of such reward‐cue salience when reward is devalued (De Tommaso et al., 2017), to see whether this unexpected and challenging finding can be accounted for by these alternative accounts (Pool et al., 2014; Watson et al., 2021). At the same time, since a different paradigm known as ‘Value Modulated Attentional Capture’ (VMAC) procedure has been developed, which would not be affected by the same confounds, a critique analysis of this paradigm is offered as well.

## TWO GENERAL PARADIGMS TO STUDY THE REWARD‐CUE ATTENTIONAL SALIENCE

Different paradigms have been proposed to address the effects of reward on visual attention and in particular to study if reward‐predicting cues capture attention as a consequence of having been paired with reward. However, despite the specific differences among paradigms, two main approaches can be identified: two‐phase and one‐phase paradigms.

### The two‐phase paradigm

This paradigm is based on a training or conditioning phase, during which a stimulus is probabilistically associated with a certain amount of reward (typically a monetary reward), followed by a test phase, when the reward cue is presented in extinction, and its ability to capture attention is measured with different tasks. An example of the two‐phase paradigm is the Value‐Driven Attentional Capture (VDAC) paradigm, originally developed by Anderson et al. (2011). In the VDAC paradigm, the initial training phase consists in a visual‐search task in which participants are rewarded for finding a colour‐defined target (red or green circle) among differently coloured circles, and for reporting the orientation of a probe line inside the target element. Correct responses are followed by monetary reward that can be high or low with different probability depending on the target colour. That is, during the training phase two colour‐defined target stimuli are associated with a high and a low reward value respectively. In a separated test phase, participants perform a task knowing that no reward will be delivered. The task consists in a visual search in which the goal is to find a shape‐defined element (a diamond) among other stimuli (differently coloured circles), and to report the orientation of the corresponding line. In a subset of trials, one of the targets of the previous task (i.e., the reward cues) appears as distractor among the non‐target elements in the array, and the attentional bias is measured as an increase in response times (RTs) caused by such distractor.

Different versions of the two‐phase paradigm have been adopted in a great wealth of studies showing the persistence of reward‐based attentional capture also with more complex stimuli and with a variety of tasks in different modalities (Anderson, 2016b), as well in the spatial (Anderson & Kim, 2018) and temporal (Raymond & O’Brien, 2009) domains of attention (for reviews see Bourgeois et al., 2016; Chelazzi et al., 2013; Failing & Theeuwes, 2017b; Luque et al., 2017).

Hence, two‐phase paradigms have been used to show that reward associations affect attention even when the cues no longer predict the reward. A test phase separated from the training phase offers also the possibility to render the reward cue completely irrelevant when their attentional salience is evaluated at test as such cues appear as distractors. Indeed, because there would be no reason to attend to previous reward cue in extinction, no strategic explanation can be invoked to interpret the corresponding attentional bias found in the test phase. Additionally, an explanation based on perceptual salience is also excluded, as in the test phase the former reward‐cues compete with equally salient stimuli for attention.

### Potential limitations of the two‐phase paradigm

Two main limitations potentially affect the two‐phase paradigm: the lingering effect of selection history, and the problematic definition of the underlying mechanisms by means of which the reward‐cue attentional salience emerges.

#### Selection history

Selection history defines the propensity to prioritize previously attended stimuli. The theorisation of such component of attentional control was introduced with the purpose of explaining growing evidence that cannot be easily accounted by the classic distinction between bottom‐up and top‐down attentional control (Awh et al., 2012; Failing & Theeuwes, 2017b). A third mechanism would rely on two types of ‘history’ that affect the current attentional selection: recent orienting episodes to an item (e.g., priming of pop‐out; Maljkovic & Nakayama, 1994), or a reward‐based past orienting experience. While the motivational component would certainly play a role in the latter, it is crucial to exclude that reward cues summon attention only because they have been attended previously.

The role of past orienting episodes, devoid of any reward component, is documented by studies showing that removing the reward delivery for the attended elements during the training task in the VDAC procedure, produces an analogous attentional bias for the same elements in the test phase (Grubb & Li, 2018; Sha & Jiang, 2016; Sha et al., 2017). That is, previously selected targets impair performance when they become task irrelevant in the test phase.

Le Pelley et al. (2016) have pointed out that selection history effects are particularly problematic for interpreting some VDAC studies (Anderson et al., 2013a, 2013b; Anderson, Laurent, & Yantis, 2012, 2014). Indeed, in the VDAC procedure the distracting effects of reward‐associated stimuli in the test phase (that served as previous targets in the training phase) are often reported as an RT difference between high‐value distractor‐present trials and distractor‐absent trials, a difference that could be due only to past orienting experience. Additionally, the effects of an unrewarded training phase on the test phase have not led to consistent results (Anderson et al., 2011; Sha & Jiang, 2016). Such inconsistency might arise because of a difference in the probe lines presented in the stimuli at test: in the VDAC studies (e.g., Anderson et al., 2011), the non‐targets elements in the display present tilted lines, but participants have to discriminate between a vertical or horizontal probe line that is displayed only in the target stimulus. Conversely, in the study by Sha and Jiang (2016) both target and non‐target elements present vertical or horizontal probe lines, a condition that might force participants to pay more attention to the shape elements to find the target, which probably made them more vulnerable to capture by the elements previously attended during training.

In order to account for selection history effects while assessing the value dependence in the VDAC procedure, Anderson and Halpern (2017) provided stringent tests showing that when reward is used in the VDAC procedure, the attentional capture triggered by previous reward cues appears to be modulated by reward value, as the previous high‐reward cues disturbs more than previous low‐reward cue. In addition, the same study showed that with an adequate sample size, an unrewarded training phase does not result in attentional capture during test. This shows that on top of any effect of selection history based only on episodes of orienting, reward history also plays an important role in modulating attentional selection (Marchner & Preuschhof, 2018). However, in the VDAC procedure the pure orienting component and the reward‐based component of selection history are not separated, and might sum‐up in determining the final effect. Therefore, the claim that attention is driven by reward value needs support from procedures in which the reward‐based component is distilled from the broaden effects of selection history.

#### Definition of the underlying mechanism

In the VDAC procedure, the cue‐reward association is formed by reinforcing the search of the cue itself. The fact that an attention bias in favour of the reward cue is found in the test phase is explained by at least two different learning mechanisms.

First, as already mentioned, because of a Pavlovian learning mechanism the motivational properties of the reward (US) transfer to the reward cue (CS), thus altering the corresponding attentional salience (Berridge, 2018; Bindra, 1978; Toates, 1986). The reward cue thus becomes an attentional magnet by means of its acquired incentive salience.

Second, according to the Stimulus‐Response (S‐R)/reinforcement system (Thorndike, 1911), during training the allocation of attention (R) toward the cue (S) would be reinforced by reward presentation. Once this S‐R association is formed, its effect will be visible for some time also in extinction (the test phase). In other words, attentional prioritisation of the reward cue would be the result of a trained action that continues even when reward prospect is absent (e.g., from the training to the test phase in the VDAC procedure). Such carry over effect of a trained action is similar to what has been referred to as an habitual orienting response instigated by reward (Anderson, 2016a; Anderson et al., 2016; Jiang & Sisk, 2019). Instrumental learning is potentially involved in every procedure in which reward is delivered contingently upon the attentional processing of the reward cue, a condition in which reward would act as a reinforcer of the attentional response. Additionally, the role of instrumental learning is particularly relevant when the task of the training phase is similar to the task of the test phase, as it happens in the VDAC procedure, where the goal is to find a stimulus in competition with other stimuli in both phases. Hence, instrumental learning can explain a bias for a previous reward cue in the test phase, as its attentional selection among other stimuli has been reinforced during training.

Both Pavlovian‐learning and instrumental‐learning mechanisms predict the same result in the context of a two‐phase paradigm (but see, Kim & Anderson, 2019), namely an attentional bias for a stimulus associated with reward. The one‐phase paradigm, conversely, would allow to distinguish between the impact of the two mechanisms.

### The one‐phase paradigm

In the one‐phase paradigm, there is no clear distinction between conditioning and testing, as the two phases are combined together. As anticipated, an example of such approach is offered by the VMAC procedure (Le Pelley et al., 2015; Pearson et al., 2015, 2016), which is essentially based on the more general omission‐contingency procedure (Sheffield, 1965). In the VMAC paradigm, participants are rewarded to search for the shape singleton in an array of irrelevant elements, and to gaze at this target element as fast as possible. In addition, one of the non‐target elements is a colour singleton, which acts as a reward cue informing participants about the amount of reward that will be gained upon the successful completion of the task. Participants, however, are explicitly discouraged to gaze at the reward‐cue colour singleton, because in this case the reward would not be obtained (omission contingency). Despite the aversive consequence of gazing at the reward cue, experiments adopting the VMAC procedure have consistently reported that participants often look at this perceptually and informative salient element (Le Pelley et al., 2015; Pearson et al., 2015, 2016). Furthermore, the rate of occurrence of such unintentional and counterproductive behaviour is proportional to the reward‐cue value: high‐value reward cues attract more erroneous saccades than low‐value reward cues (Le Pelley et al., 2015; Pearson et al., 2015, 2016). In agreement with previous findings obtained with the same paradigm, Watson et al. (2021) have recently found a stronger oculomotor bias for a valued reward‐cue than for a devalued one.

The VMAC procedure is meant to overcome the methodological limitations discussed previously, namely that reward cues summon attention because of strategic factors, of broad effects of selection history, or as a result of instrumental learning (Le Pelley et al., 2015). Since in the VMAC procedure the cost incurring for looking at the reward cue is made explicit to participants, this would guarantee that the nature of the cue attentional bias observed, if any, is not due to a strategic/voluntary allocation of attention to the reward cue. Rather, such bias would be caused by an involuntary allocation of attention triggered by the cue motivational salience. Likewise, the results obtained with the VMAC procedure would not be affected by selection history (Anderson & Halpern, 2017; Awh et al., 2012; Failing & Theeuwes, 2017b), namely by the fact that the reward cue gains attentional salience because it has been repeatedly selected during the task. Here, the argument is that because in VMAC the reward‐cue is never the target of the task, then it is never voluntarily attended, and therefore no selection history can develop, as instead would happen in other paradigms (see VDAC procedure). Finally, and most importantly, the VMAC procedure would allow to disentangle the role of Pavlovian learning from that of instrumental learning in generating the reward‐cue attentional salience. While Pavlovian learning predicts that the high‐value reward cue will attract *more* erroneous saccades because it is associated with a higher motivational value, the instrumental‐learning hypothesis would predict the opposite, as the action to search for the reward cue is never reinforced. In fact, it is the action of withdrawing attention from the reward cue that is reinforced. Instrumental learning would predict, therefore, the development of a learned attentional suppression response (Della Libera & Chelazzi, 2009) that will result in *less* erroneous saccades to the high‐value reward cue. Results from the VMAC procedure clearly attribute to Pavlovian learning the origin of the reward‐based attentional bias, as high‐value reward cues attract more erroneous saccades than low‐value reward cues (Le Pelley et al., 2015; Pearson et al., 2015, 2016).

### Potential limitations of the one‐phase paradigm

Let us now consider to what extent the VMAC procedure is really immune from potential confounds. In particular, we should carefully ponder whether in this paradigm attention is summoned by the reward‐cue distractor only because of its motivational salience gained in virtue of the Pavlovian association with reward. According to the studies in which this paradigm has been proposed, an automatic value‐driven capture explains the unwanted saccades toward the reward‐cue distractor. Such oculomotor behaviour would be completely non‐strategic because it is never rewarded, as saccades toward the cue are followed by reward omission. While the motivational salience of the reward‐cue distractor can certainly contribute to such oculomotor behaviour, it should be noted that in the VMAC paradigm the reward‐cue is also a colour singleton, namely the most salient item in the array, and since the original study of Theeuwes (1991) it is well established that colour singletons capture attention irrespective of any top‐down set or strategic factors (also see Turatto & Galfano, 2001). Furthermore, it has been shown that a colour singleton can trigger an unwanted oculomotor capture irrespective of any reward (Theeuwes et al., 2003). Despite in the VMAC task, the oculomotor capture is value dependent (i.e., high‐reward cues draw more saccades that low‐reward cues), it appears difficult to argue that such oculomotor capture in the VMAC paradigm is only triggered by the reward‐cue motivational value. In addition, the attentional capture component explained by the reward cue perceptual salience causes a repeated, albeit involuntary, selection of the distractor, which does not rule out a possible contribution of the selection history account.

Another issue worth considering is that although participants know in advance that reward will be omitted when a saccade is made to the cue, it is difficult to consider such cue as ‘irrelevant’ for the task. If the cue is irrelevant to perform the task, it is not irrelevant for the consequence of the task performance, as it provides important information concerning what would be gained if the correct response is provided. In other words, in addition to its perceptual salience the reward cue is likely to attract attention because it is highly informative of the outcome. The role of the informative component of the reward cue is a critical factor that we will discuss more thoroughly in relation to the consequences of the attentional processing of the cue, and in terms of information seeking behaviour. For now, we want to stress the fact that the reward cues in the VMAC task, as already argued by Anderson and Halpern (2017) and Bucker and Theeuwes (2017), can hardly be considered to be irrelevant. Therefore, it appears that it cannot be excluded that the oculomotor capture behaviour reported in the VMAC procedure can be at least partially explained either by the informative nature of the cue and/or by the it is perceptual salience, with both factors modulating the motivational salience of such reward cue.

## OVERCOMING POTENTIAL LIMITATIONS: ALTERNATIVE PROCEDURES

In the attempt to devoid the reward cue of its informational relevance in the VMAC procedure, a recent study by Watson et al. (2019) has completely removed the reward delivery in the last two blocks of trials, thus making the reward cues completely irrelevant. Despite participants were informed of this manipulation, the oculomotor bias for the reward cues did not vanish even during the last unrewarded blocks. The study by Watson et al. (2019) is a first step toward a procedure that combines the single and the two‐phase procedures described previously.

Anderson and Halpern (2017) have already noted that a single‐phase VMAC‐like procedure and two‐phase procedures both present strengths and weaknesses, and proposed that a hybrid procedure, combining the VMAC task for the associative training phase, with a reward‐free test phase, might provide some advantages in separating motivational influences from selection history. As a matter of fact, such hybrid procedure was used by De Tommaso et al. (2019; Experiment 3) to determine the motivational nature of the attentional bias emerged in previous experiments. In this experiment, a VMAC‐like procedure, as described by Le Pelley et al. (2015), was implemented in the associative phase, and a separated visual search task was used in the subsequent test phase. The visual‐search task implemented in De Tommaso et al. (2019; Experiment 3) was quite different from the one typically used in the two‐phase paradigms (e.g., Anderson et al., 2011), and this because all the former reward cues appeared simultaneously in the search display, each one carrying its own associated value, and each one containing a letter. There was one target letter (T), and other non‐target letters (L), and participants had to report the orientation of the target. Crucially, the letter T had the same probability to occur in each element of the display, which made the previous reward cue completely irrelevant. Therefore, this visual‐search task differs in one important aspect with respect to the standard visual‐search task used in previous VDAC procedures (e.g., Anderson et al., 2011), where the target of search is a shape singleton, and the previous reward cue, acting as distractor, is one of the remaining non‐target shapes. In the standard visual‐search task used in previous VDAC procedures, each element of the display contains an oriented line, and the task is to report the orientation of the probe line inside the target shape. Under these conditions, the defining features of the elements of the display need to be processed to find the target, which may explain why the previous reward cue captures attention as a function of its association with reward. By contrast, in De Tommaso et al.’s paradigm the target is the letter T, which has nothing in common with the surrounding elements made of the previous reward cues, which can be totally ignored to find the target. However, despite this total independence between the features of the target with those of the distractor, the latter still captures attention because of its acquired motivational salience.

The results obtained by De Tommaso and colleagues showed an attentional bias proportional to the reward‐cues associated value, as the target letter was discriminated faster when it appeared inside the previous higher‐value reward cue. With the effects of a VMAC task measured in a separated and unrelated test phase administered in extinction, alternative interpretations of attentional capture, such as a strategic attentional allocation and the effects of selection history, become much less plausible to explain the results reported by De Tommaso et al. (2019; Experiment 3).

However, because in the VMAC procedure adopted in De Tommaso et al.'s (2019; Exp. 3) study the reward cue was a salient and informative item, it could still be argued that the results found in the separate visual search were due to a lingering effect of a repeated attentional capture triggered by the reward cue during the training VMAC‐like phase (Theeuwes, 1991; Theeuwes et al., 2003). By these means, uncertainty remains about the role of instrumental learning in explaining the attentional bias documented in De Tommaso et al.’s study. Indeed, instrumental learning could still play a role as each cue during conditioning might have captured attention because of its salience and informative value, and this attentional response was reinforced.

To address the perceptual salience issue, Failing et al. (2015) administered a VMAC‐like procedure in which the search display was similar to the one used in Le Pelley et al.’s (2015) study, with the critical exception that the reward cue was not a colour singleton. Participants were informed about the specific colours that signalled the high or low reward, and informed that reward would have been omitted if they had looked at the reward cue. Nevertheless, more oculomotor capture was found for the high‐value reward cue relative to the low‐value reward cue, which suggests that the perceptual salience of the reward cue is not sufficient to explain the counterproductive oculomotor capture it triggers. It is worth noting, however, that informing participants about the colours that signalled reward, and by asking them at the same time to ignore such singleton element, might have induced a sort of ‘don't think of the white bear’ attentional effect, whereby attention is paradoxically allocated to the information that needs to be ignored, which may have made the irrelevant stimuli salient. However, despite participants were informed that the colour of the singleton element indicated the amount of reward, they were not informed about the specific colour‐reward magnitude contingency. By these means, participants had to figure out such contingencies by themselves. An ‘attentional white bear’ effect might have developed once a colour‐reward magnitude information was extracted, but the same can be argued for other experiments in which a high‐ versus low‐reward associated stimuli produced a different attentional bias. More recently, Pearson et al. (2020) tried to rule out the role of the cue perceptual salience in the VMAC task by using a display that forced participants to adopt a specific attentional set to find the target, which should increase the possibility to ignore the salient cue. The rationale stems from evidence suggesting that there are conditions in which salient stimuli can be ignored as experience with the stimulus develops, and because of the adoption of a particular attentional strategy during search (Bacon & Egeth, 1994; Gaspelin & Luck, 2018; Vatterott & Vecera, 2012). In particular, when searching for a unique element in the array (for example a shape singleton), attention is set to detect a discontinuity in the display (*singleton*‐*detection mode*), so the presence of another singular element (for example, a colour‐singleton) will likely attract attention. In other words, the attentional templates of the relevant and the irrelevant information overlap, being both tuned on singularity. However, if attention is tuned to a more specific feature of a target (*feature*‐*search mode*), the strategy of searching for a singleton becomes inefficient, and the distractor singleton can be ignored with practice (Gaspelin et al., 2017). Therefore, learning allows to inhibit distracting singletons in the array, leading to habituation of the orienting response (De Tommaso & Turatto, 2019). Because an appropriate search strategy can be induced, Pearson et al. (2020) reasoned that with the adoption of a feature‐search mode in the VMAC procedure the oculomotor capture by reward cues should not be attributed to its physical salience. To implement the idea, the search display was composed by elements of different shapes, while the reward‐cue remained a colour singleton. The results showed that the high‐value reward cue attracted more counterproductive saccades, and lead the authors to conclude that the attentional capture by reward cues overcomes inhibitory suppression.

On the other hand, other studies have tried to address the role of task relevance in affecting the salience of reward cues. As anticipated, one problem of interpreting the attentional grabbing power of reward cues as the result of Pavlovian learning is that they remain task‐relevant stimuli because they are informative about the reward obtained in a given trial for a correct response. It appears, therefore, that there are two components of a reward cue that need to be dissociated. The first is the *informative component*, namely the fact that the cue anticipates the possible reward gain (see Pearson & Le Pelley, 2020). The second is the *instrumental component*, or the stimulus‐response relation with reward in terms of the consequences of attending to that stimulus. It should be noted that in the procedures reviewed so far, the informative component and the instrumental component of reward cues act in concert, although they might produce either additive or subtractive effects on attention. One study that sought to isolate the role of the informative component is that of Bucker and Theeuwes (2017). In this study, which was structured as a two‐phase procedure, participants were presented with a series of figures in which a colour‐singleton signalled a high or low reward depending on its colour. Importantly, reward delivery was purposely not contingent to participants’ task performance, which was to detect a change in the fixation cross. In this way, the instrumental component of the reward cue was excluded, as attending to the cue did not entail any consequence (neither reward nor omission). A test phase, based on the additional singleton paradigm (Theeuwes, 1992), showed that high‐value reward cues distracted more than the low‐value reward cues. In a more recent study Bucker and Theeuwes (2018) replicated their findings with a conditioning phase in which the reward cue was not salient, because it appeared in competition with another equally salient stimulus. Indeed, if a reward cue is the most salient item in the display, a problem could arise because it could draw attention even if it carries no instrumental relation with reward, and the reinforcement of such attentional orienting could transfer in the visual search test phase. Altogether, these results highlight the informative component of reward on attention, by excluding a possible influence of the instrumental component. These studies, therefore, corroborate the importance of Pavlovian learning as the mechanism underlying reward‐cues attentional capture.

The relationship between the informative component and the instrumental component of reward cues was not directly addressed in the studies reviewed so far, as both components either coexisted or one was simply excluded from the design (as in Bucker & Theeuwes, 2017, 2018). With this regard, it is worth recalling the logic behind the development of the VMAC procedure, which has been devised to create the conditions in which the Pavlovian mechanism and the instrumental mechanism make opposing predictions. It should be emphasised that results supporting the Pavlovian mechanism does not demonstrate its exclusive effect on attention, as the instrumental effect could still play a role that under certain conditions may be overshadowed. Accordingly, one recent study by Pearson and Le Pelley (2020) attempted to measure the contribution of the instrumental component over the informative one (also see Harris et al., 2013). In this study, a standard VMAC procedure was administered to a group of participants. As noted, in this procedure the informative component and the instrumental component of reward cues coexist, in that the cue is informative about the upcoming reward, and it is followed by certain consequences when looked at (i.e., reward omission). Crucially, another group of participants experienced the VMAC task without the instrumental component, as they were presented with the same reward schedule in a ‘yoked’ version matching the other group of participants. That is, this ‘yoked’ group experienced reward delivery independently of whether or not they looked at the reward cue. Both groups showed the oculomotor capture driven by the reward‐cue value, but the effect was significantly smaller for those who experienced a response‐dependent reward omission. Thus, it appears that in the VMAC procedure the informative and the instrumental component act in concert to confer attentional priority to the reward cue, with the former increasing attentional priority and the latter diminishing it.

The study by Kim and Anderson (2019) represents another attempt to unravel the interplay between the instrumental component, the Pavlovian component and selection history. By means of a two‐phase paradigm, the study showed that participants became more efficient in producing antisaccades for stimuli followed by high reward, highlighting the effect of the instrumental component on the attentive behaviour. However, such high‐value stimuli attracted attention in a following test phase in which a prosaccade toward the same stimuli was required, an effect attributable to a Pavlovian component. In a second experiment, the reward was omitted, and selection history was investigated by modulating the frequency of stimuli appearance, as participants were trained to make an antisaccade to frequent or infrequent colour‐defined stimuli. The test phase showed a habitual tendency to avoid frequent stimuli as compared to infrequent stimuli, demonstrating that past orienting experience plays a role that operates independently of reward history, and in some contexts can produce opposite effects.

Another way to disentangle the contribution of reward and selection history is to examine the contextual dependence of the induced attentional bias. With this regard, evidence shows that while value‐driven attentional bias is context specific (Anderson, 2015), the effects of selection history generalise through different contexts (Anderson & Britton, 2019), suggesting that reward and selection history may affect attention through different mechanisms. Therefore, changing the context between training and test settings could help to define the nature of the attentional bias measured, which in case of selection history should persist in a new context.

To further complicate the scenario, it was noted that the instrumental learning mechanism might not be related solely to the instrumental component of reward cues. Rather, instrumental learning could affect also the informative component of the reward cues. That is, instrumental reinforcement is not only limited to the contingency of attentional processing and reward delivery. Instrumental reinforcement could arise also by the intrinsic rewarding properties of the informative component of reward cues, as the information‐seeking behaviour hypothesis assumes that the information that is provided by the reward cues could be rewarding *per se* (Bromberg‐Martin & Hikosaka, 2009; Kobayashi & Hsu, 2019). As it was reported in Watson et al.'s (2019; p. 9) study about the VMAC task, ‘…an alternative possibility is that […] participants learn that looking at the high‐reward distractor provides useful information (that a high reward is available), and this gain in information provides reinforcement for the conditioned, instrumental response of attending to the high‐reward distractor’. While so far this remains a mere speculation, this possibility blurs the definition of what could reliably be considered a Pavlovian learning in the context of attentional salience of reward cues. With this regard, to isolate the role of Pavlovian learning, instrumental learning can be excluded by an appropriated procedural design, as previously reported (Bucker & Theeuwes, 2017, 2018; H. Kim & Anderson, 2019; Pearson & Le Pelley, 2020). However, excluding the instrumental component may not be sufficient to isolate the effects of Pavlovian learning, because also the remaining informative component can involve an instrumental learning mechanism. Therefore, to exclude the role of any instrumental learning mechanism one should eliminate also the informative component. However, if both the informative and the instrumental components of the reward cues are eliminated, one may question the ontological nature of the reward cue itself, wondering whether a reward cue that is neither informative nor in a contingent relation with reward could even possibly exist.

## DYNAMIC CHANGES IN THE REWARD‐CUE ATTENTIONAL SALIENCE

While the idea that the reward‐cue attentional salience is modulated by the associated reward value is widely accepted, a few studies have investigated whether such salience is updated when the reward value changes. As already argued previously, this question appears to be crucial as the motivational value of a given reward is not fixed, but fluctuates in accordance with the corresponding drive. Therefore, as much as a slice of pizza is highly valuable and desirable when we are hungry but becomes unattractive after lunch, the signboard ‘Pizzeria’ is a reward cue that can attract our attention if we are starving, but that can go unnoticed when we are sated. Motivated by these observations, a small and perhaps overlooked line of research has begun to investigate the interaction between the attentional salience of a reward cue and the dynamic change of reward value (De Tommaso et al., 2017; De Tommaso & Turatto, 2021; Pool et al., 2014; Watson et al., 2021). The novelty of these studies is the adoption of a type of reward that can be easily devalued. Most studies investigating the effects of reward on human cognition have used pecuniary rewards with the assumptions that the desirability of this reward remains constant in the experiment independently of how much reward has been gained. Conversely, primary rewards (such as drinks) are desirable as long as the corresponding physiological state is relatively high (e.g., the agent is thirsty), and cease to be desirable when the physiological state is relatively low (e.g., the agent has quenched his thirst). By adopting primary rewards, it is possible to ascertain whether the cue attentional salience is updated when the reward value changes.

Stemming from these observations, the study of Pool et al. (2014) has reported that cues associated with a chocolate odour speeded up the attentional orientation in a spatial cueing task, a result that parallels the outcome obtained with monetary reward (Failing & Theeuwes, 2014). Crucially, Pool and colleagues altered the reward value by allowing some participants to eat chocolate at will after conditioning, and found no modulation of the reward cues on the spatial cueing task, suggesting that the reward cue salience decreases automatically with reward devaluation.

Another series of studies (De Tommaso et al., 2017; De Tommaso & Turatto, 2021) have reported a quite different scenario. By using drink rewards delivered to thirsty participants, it was found that previous reward cues persisted to capture attention in a subsequent visual‐search task despite participants quenched their thirst after the initial conditioning phase. These results suggest that the salience of the reward cue can outlast reward devaluation, and does not update automatically when the value of the associated reward changes. Furthermore, such irrational reward‐cue attentional bias persisted for a week after conditioning, and was unaffected by a new incentive learning (Balleine, 1992; Dickinson & Balleine, 1994). However, a dynamic change in attentional capture by the reward cue was obtained only when a new learning of the cue‐reward relation developed in condition of high physiological state, namely when participants where thirsty. That is, the modification of the acquired attentional salience of a reward cue appears possible only during a new high motivational state relative to reward (De Tommaso & Turatto, 2021). Accordingly, a similar modification of the attentional bias, although not explicitly tied to biological needs, was also reported in a recent study by Liao and Anderson (2020) where cue‐reward contingencies were reversed during a new learning phase. The study documented that the attentional bias eventually conformed to the new cue‐reward mapping, although the modification was not immediate, leading the authors to suggest that value‐based attentional priority has a sort of inertia in updating to new reward contingencies.

Clearly, the results from Pool et al. (2014) and De Tommaso et al. (2017) seem incompatible. Namely, according to the study of Pool et al. (2014) the attentional salience of reward cues adapts to changes (e.g., devaluation) in reward value, whereas this flexibility is severely limited according to the study of De Tommaso et al. (2017). Such differences are not easy to reconcile, but it may be speculated that the different methods used in the abovementioned studies may be attributed to a different degree of sensitivity of the two paradigms in detecting motivational effects on attention. In the study by Pool and colleagues, during the conditioning phase participants were required to press a bar to unmask a CS+ that was associated with a chocolate odour, or a CS−, which was associated with an odourless air. Hence, the conditioning phase did not require participants to be actively involved in processing the CS‐reward relation. When the CS+ and CS− were then used in a spatial cueing task after conditioning, the CS+ speeded up performance compared to the CS−. However, this beneficial effect of the CS+ was not present in a group of participants that ate chocolate at will between conditioning and test. By contrast, in De Tommaso et al.’s study during conditioning participants had to maximise reward by actively exploring the cues relation with reward. Such difference in establishing the cue‐reward relation might have played a role in the attribution of attentional salience, although at present this possibility remains only a speculative hypothesis.

At this point, one may wonder whether a persistent reward cue attentional bias could be explained by alternative accounts. First of all, it is worth recalling that the paradigm used by De Tommaso et al. (2017) is an example of a two‐phase procedure, in which a conditioning phase is followed by a test phase, where a visual‐search task is performed in extinction. One could argue that the conditioning procedure that has been adopted may be problematic, as participants responded to different cues as a function of the reward associated probability. Specifically, the attentional bias emerged in favour of the previous best‐predicting reward cue, would not be due to an associative link between cue and reward, but because during conditioning the more a cue was predictive of reward the more it was attended and responded to (Failing & Theeuwes, 2017a, 2017b; Sha & Jiang, 2016). However, if this were the case, namely if the attentional bias were only driven by the past selection of the reward cue; then, it should not be affected by reward value. Conversely, if the attentional bias does indeed reflect a transfer of the reward motivational properties to the cue during the associative phase, then by removing the reward motivational value before the associative phase, the reward cues should not gain any attentional salience, and no attentional bias should emerge during visual search in the test phase. Experiment 3 in De Tommaso et al.’s (2017) study was specifically designed to tackle this issue, and the results showed that the reward‐cue attentional bias at test disappeared when participants were sated before the conditioning phase, despite in this phase the rate of responses to reward cues was different. This attentional bias is quite reliable, as it has been replicated in another recent study (De Tommaso & Turatto, 2021), and indicates that the reward‐cue attentional bias reported in the test phase was driven by motivation. As a matter of fact, De Tommaso et al.’s findings anticipated what has been recently reported by Watson et al. (2021) with a different approach, namely that if an outcome does not have value when it is associated with the reward cue, the latter does not develop attentional salience.

The second issue to be examined concerns the putative role of selection history in accounting for De Tommaso et al.’s (2017; also see De Tommaso & Turatto, 2021) findings. As mentioned before, the idea is that the attentional bias for a particular stimulus may develop as a result of the repeated attentional selection of that stimulus. With this regard, and as discussed elsewhere (De Tommaso et al., 2019), one may note that in the procedure of the De Tommaso et al. (2017) study the reward‐cue attentional selection was equalised across the different reward cues during the associative phase. Indeed, each cue, regardless of its predictivity, needed to be attended equally by participants in order to decide whether or not to respond, given that the overall number of responses available was limited (for more details see De Tommaso et al., 2017). Moreover, during conditioning each cue neither was searched for nor competed for attention, given that on each conditioning trial the cue was the only element that abruptly appeared on the screen. It follows that the action of searching for a particular stimulus, similarly to what was required in the visual‐search task at test, was not reinforced during conditioning, and consequently, the resulting attentional bias cannot be interpreted as a conditioned visual‐search response for the reward cue.

However, it has to be acknowledged that at variance with the VMAC procedure, in De Tommaso et al.’s (2017) study the cue was the target of the task during the conditioning phase; therefore, one could argue that the attentional bias emerged in the test phase is not necessarily the product of a Pavlovian mechanism, but instead of an habitual orienting response instigated by reward. In other words, in De Tommaso et al.’s (2017) study the attentional bias would have been the product of the reinforcement of the attentional orientation to the reward cues, a criticism that would affect other procedures previously discussed. To put things in a different perspective, there is no doubt that in De Tommaso et al.’s (2017) studies the informative component and the instrumental component of reward cues coexist during conditioning, as reward cues convey information about the probability of reward occurrence and reward is contingently delivered upon the attentional processing of the cue. As already discussed, these conditions favour instrumental learning, which in the case of the De Tommaso et al. (2017) procedure would concur with Pavlovian learning in determining the attentional priority of the reward cue. Instrumental learning would arise from the repeated processing of the reward cue, and from the reinforcement of such attentional selection (albeit only after a response is emitted). The Pavlovian mechanism, in parallel, would confer motivational salience to reward cues by repeated association with reward. Once established that the role of both processes is at play, an interpretation of the results of De Tommaso et al. (2017) based solely on Pavlovian conditioning is unwarranted.

One way to overcome the confound of a differential reward‐cue response selection is suggested by a recent study by Qin et al. (2021), investigating how reward affects the perception of its predictors at a low‐level visual features. The study adopts a conditioning phase in which participants responded to the cues depending on their predictive value. Specifically, participants had to choose between pairs of a high‐value reward cue vs. no reward cue, or low‐value reward cue vs. no reward cue. In this way, response choice of high and low‐value reward cues should be paired, because both would be chosen over the no reward cue. The study reports an attentional bias for the high‐value reward cue despite responses to the low‐value reward cue was paired, suggesting that the role of response choice is not sufficient in explaining the motivational effect on attention.

Despite the procedures adopted in De Tommaso et al. (2017, 2019) can reflect a role of both Pavlovian and Instrumental learning, it seems safe to exclude the contribution of strategic factors, and of selection history as far as the mere orienting of attention is concerned regardless of any motivational factor. Hence, it can be concluded that showing that the reward‐cue attentional salience persisted despite reward devaluation remains a novel and unexpected finding revealing new interactions between attention and motivation.

At this point, it is useful to consider what previous studies using the VMAC paradigm can tell us about dynamic changes in the reward‐cue attentional salience. For example, let us consider the recent study from Watson et al. (2021), whose results can be explained by a Pavlovian learning mechanism. The study showed both the effects of associating a reward cue with both a valued (hungry participants) and a devalued food reward (sated participants), with the reward cue failing to gain any attentional salience in the latter condition. The latter finding is expected because the food outcome was consumed before the associative phase, and therefore there was hardly any motivational property left to transfer from the outcome to the cue during conditioning. However, the study does not test whether the attentional capture elicited by a reward cue changes as a function of the reward value, for there was no change in the outcome value after the cue‐outcome association. In other words, since reward was devalued before the conditioning phase, it was impossible for Watson et al. (2021) to establish whether the salience of a reward cue dynamically updates according to variations in reward value. From this point of view, the results are neither in accordance with those of Pool et al. (2014) nor at odds with those of De Tommaso et al. (2017), as in these studies the reward cue was first associated with an highly desirable outcome, and then the outcome was devalued and the attentional bias measured. Hence, the question regarding the dynamic link between attentional bias and motivational shifts was not properly addressed by the study of Watson et al. (2021).

It has to be acknowledged, however, that in principle the VMAC procedure can be used to study dynamic changes in the reward cue value, but this requires to make the VMAC procedure a two‐phase paradigm, in which reward is devalued after the initial training.

## CONCLUSIONS

Even though compelling evidence suggests that reward cues gain attentional salience via Pavlovian learning (Bucker & Theeuwes, 2017, 2018; Le Pelley et al., 2015), still alternative accounts need to be to ruled out when a given procedure is used to investigate this process. Indeed, the lingering effects of the past orienting episodes could favour the formation of an attentional bias that might jointly operate with, or conversely counteract, the motivational effects of reward (Failing & Theeuwes, 2017a; Kim & Anderson, 2019; Pearson & Le Pelley, 2020).

Investigating dynamic changes in the reward‐cue attentional salience induced via alteration of physiological needs (e.g., thirst or hunger) could help in disentangling the learning mechanisms whereby the reward‐cues attentional bias is attained. Recent studies confirm that the reward‐cues attentional salience adapts, although with some inertia, to changes in cue‐reward mapping when reward is a desirable outcome (De Tommaso & Turatto, 2021; Liao & Anderson, 2020; Watson et al., 2021), but, on the other hand, provide indications that, once established, it does not follow changes in reward value (De Tommaso et al., 2017; De Tommaso & Turatto, 2021; but see, Pool et al., 2014). Interestingly, the fact that the reward cue attentional salience resists to update according to reward value is in agreement with studies showing a dissociation between the motivational effects of reward cues and those of the reward itself that has emerged in the domain of controlled behaviour (De Tommaso et al., 2018; Pool et al., 2015, 2019).

It is also worth noting that individual differences in susceptibility to attentional capture by motivational stimuli such as personality traits (Albertella et al., 2020), sensation seeking (Hickey et al., 2010), age (Roper et al., 2014) and attention disorders (Sali et al., 2018) may also influence the ability to adapt, or conversely be resilient, to dynamic changes in motivation, and these factors should be taken into account in future investigations. Accordingly, it was recently reported that an induced state of anxiety can reduce the magnitude of the attentional bias to reward‐related stimuli (Kim & Anderson, 2020), suggesting a state‐dependent modification of reward‐related attentional capture. In addition, similarities and differences have emerged between reward‐ and threat‐associated stimuli in the control of attention (Anderson & Britton, 2020; Britton & Anderson, 2021; Nissens et al., 2017).

In sum, different procedures investigating reward‐cues attentional salience have been devised, highlighting several factors that need to be controlled in order to infer that attention is biased by the motivational effects of reward. Among these factors, past orienting episodes of selection or avoidance, reinforcement of the attentive behaviour and information seeking have shown to be particularly relevant and not easy to disentangle. However, several strategies have been implemented to control for the role of these factors, such as for example to test whether the attentional bias is context specific, or whether the presence of the attentional effect persists despite the removal of reward from the learning procedure. Furthermore, studying the role of state‐dependency in modulating the reward‐cue salience, as a consequence of either dynamic change in motivation or of an altered arousal level, could also offer new insights on how reward‐cues attain and maintain their capacity to attract attention.

## CONFLICT OF INTEREST

The authors declare that they have no conflict of interest.

## DATA AVAILABILITY STATEMENT

Data sharing not applicable to this article as no datasets were generated or analyzed during the current study.
